# In vitro potency of amikacin and comparators against *E. coli,K. pneumoniae* and *P. aeruginosa* respiratory and blood isolates

**DOI:** 10.1186/s12941-016-0155-z

**Published:** 2016-06-17

**Authors:** Christina A. Sutherland, Jamie E. Verastegui, David P. Nicolau

**Affiliations:** Center for Anti-Infective Research and Development, Hartford Hospital, 80 Seymour Street, Hartford, CT 06102 USA; Division of Infectious Diseases, Hartford Hospital, Hartford, CT USA

**Keywords:** *Amikacin*, *E. coli*, *K. pneumoniae*, *P. aeruginosa*

## Abstract

**Background:**

The purpose of this study was to define the potency of amikacin and comparator agents against a collection of blood and respiratory nosocomial isolates implicated in ICU based pulmonary infections gathered from US hospitals.

**Methods:**

Minimum inhibitory concentrations of amikacin, aztreonam, cefepime, ceftazidime, ceftolozane/tazobactam, ceftriaxone, ciprofloxacin, imipenem, meropenem, piperacillin/tazobactam and tobramycin were tested against 2460 Gram-negative isolates. Amikacin had 96 % susceptibility against the combined *E. coli* and *K. pneumoniae* isolates and 95 % susceptibility against *P. aeruginosa*.

**Results:**

Ninety-six percent of all of isolates tested were susceptible (i.e., MICs ≤16 mg/L) to amikacin by current laboratory standards which demonstrates a high level of activity to combat infections caused by these organisms including ESBL, MDR, β-lactam and fluoroquinolone resistant strains. Moreover, 99 % of all organisms had amikacin MICs ≤64 mg/L.

**Conclusions:**

Overall, these data highlight the continued potency of amikacin and suggest that the achievable lung concentrations of approximately 5000 mg/L with the administration of the amikacin by inhalation (Amikacin Inhale, BAY41-6551) will exceed the MICs typically observed for *P. aeruginosa, E. coli* and *K. pneumoniae* in the hospital setting.

## Background

The management of hospital-acquired pneumonia (HAP) and ventilator-associated pneumonia (VAP) has been made increasingly difficult due to the emergence of resistance and the potential for reduced antibiotic lung penetration in the intubated patient. HAP continues to be the second most common cause of nosocomial infections in the United States and is associated with increases in hospital length of stay, healthcare costs and represents a major cause of mortality especially in critically ill patients [1, 2]. VAP, a subset of HAP, that occurs in mechanically ventilated patients more than 48 h after tracheal intubation and occurs in 9–40 % of mechanically ventilated patient’s making it among the most frequent infections in the ICU [1, 3].

Gram-negative bacteria are responsible for a substantial proportion of HAP and VAP infections. *Pseudomonas aeruginosa,* along with the Enterobacteriacae, *Escherichia coli* and *Klebsiella pneumoniae* are amongst the most common etiological organisms representing approximately two-thirds of causative agents [4, 5]. Nosocomial pneumonia caused by *P. aeruginosa*, *E. coli* and *K. pneumoniae* continues to pose significant challenges in US hospitals due to their prevalence and the acquisition of numerous antimicrobial resistance mechanisms. As a result, the selection of empirical antibiotic therapy in patients with nosocomial respiratory tract infections has become increasingly challenging as the number of potentially effective agents has been reduced due to evolving resistance. Yet more challenging is delivering sufficiently high antibiotic concentrations to the lung as many parenteral therapies have poor or variable penetration. The delivery of antibiotics directly to the site of infection presents a unique clinical opportunity to enhance patient outcomes by achieving high local concentrations that overcome resistance while minimizing the potential for toxicity associated with systemic administration.

Amikacin Inhale (BAY41-6551) is a reformulated solution of amikacin (AMK) combined with a drug-delivery module that is currently under phase III study as an adjunctive therapy for the treatment of Gram-negative pneumonia in intubated and mechanical ventilated patients. In vitro pharmacodynamic models evaluating the achievable epithelial lining fluid (ELF) concentrations after the administration of AMK inhalation against Gram-negative organisms demonstrated rapid and sustainable bactericidal killing of AMK both alone and in combination with systemic exposures of meropenem when AMK MICs were ≤256 mg/L [6]. Our objective was to define the potency and MIC distribution of AMK against a US collection of *P. aeruginosa*, *E. coli, K. pneumoniae* nosocomial isolates and relate these data to achievable lung concentrations of the compound when delivered via the aerosol route.

## Methods

Fifty US hospitals, 41 teaching and 9 community provided non-duplicate nosocomial blood and respiratory isolates of *E. coli, K. pneumoniae* and *P. aeruginosa* from adult inpatients. Additionally, five of these hospitals also provided *S. maltophilia* respiratory isolates. Organisms were identified at each participating site using the standard methods. The isolates were transferred to trypticase soy agar slants for shipping to the Center for Anti-Infective Research and Development, Hartford Hospital, Hartford, CT, USA. Collection occurred from 2013 into 2014.

Clinical Laboratory Standards Institute (CLSI) defined broth microdilution methods were employed to determine minimum inhibitory concentration (MICs) for AMK, aztreonam (ATM), cefepime (FEP), ceftazidime (CAZ), ceftolozane/tazobactam (C/T), ceftriaxone (CRO), ciprofloxacin (CIP), imipenem (IPM), meropenem (MEM), piperacillin/tazobactam (TZP) and tobramycin (TOB) [7]. Antibiotics were purchased from Sigma (St. Louis, MO) except for C/T which was provided by Cubist Pharmaceuticals. Quality control was performed on each batch of MIC testing using *E. coli* 25922 and *P. aeruginosa* 27853 as defined by CLSI. All transfer and colony counts were performed on trypticase soy agar plates containing 5 % blood. CLSI and FDA breakpoints were used to define susceptibility. For C/T the FDA breakpoints of 2 mg/L for Enterobacteriaceae and 4 mg/L for *P. aeruginosa* were utilized [8]. Isolates that were non-susceptible to AMK (i.e., ≥32 mg/L) by current laboratory definitions were repeated and confirmed.

*Pseudomonas aeruginosa* were identified as multidrug resistant (MDR) if they displayed resistance to 3 or more classes as represented by the following phenotypic resistance profiles: CIP (MIC ≥4 mg/L), IPM (MIC ≥8 mg/L), CAZ (MIC ≥32 mg/L), TZP (MIC ≥128 mg/L) and TOB (MIC ≥16 mg/L) [9].

*Escherichia coli* and *K. pneumoniae* were tested for extended spectrum β-lactamases (ESBL) production if they had an MIC of ≥1 to 2 mg/L of the following: ATM, CRO or CAZ. CLSI defined ESBL confirmation studies were then undertaken using additional MIC testing with CAZ, CAZ with clavulanate, cefotaxime and cefotaxime with clavulanate [7].

Isolates testing non-susceptible to ertapenem, imipenem, or meropenem were evaluated for carbapenemase production using the CarbaNP test [10].

## Results

**Fig. 1:**
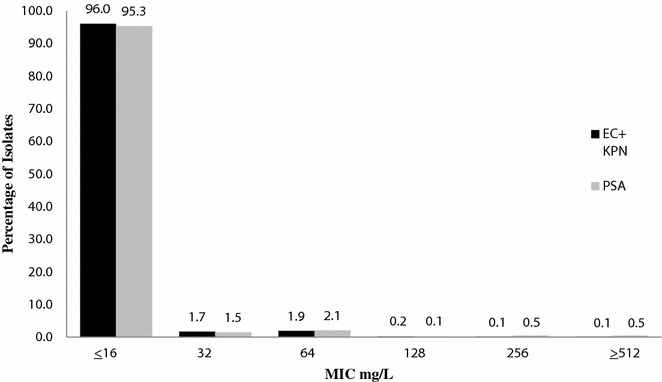
Distribution of Amikacin (AMK) against *E. coli* (EC) and *K. pneumoniae* (KPN) versus *P. aeruginosa* (PSA)

Hospitals provided nosocomial blood (n = 1118) and respiratory isolates (n = 527) of *E. coli* (n = 811) and *K. pneumoniae* (n = 835). Demographic from these patients are shown in Table 1. Rank order % susceptibility for the Enterobacteriaceae, was as follows: AMK 96 %, IPM 96 %, MEM 96 %, C/T 94 %, TZP 88 %, FEP 87 %, CAZ 85 %, ATM 85 %, TOB 84 %, CRO 84 % and CIP 74 % (Table 2). For AMK 96 % of these organisms had MICs ≤16 mg/L, 2 % 32 mg/L and 2 % ≥64 mg/L (Fig. 1). Comparing AMK against both FEP (n = 216) and TZP resistant (n = 191) isolates only 2 % of the organisms had MICs ≥128 mg/L, respectively. Interesting one *E. coli* isolate was found to have an AMK MIC of 65,536 mg/L. Isolates from blood had a slightly higher (1–7 %) susceptibility for all agents tested than the respiratory isolates.

**Table 1:** Age, hospital location and infection site of patients with *E. coli*, *K. pneumoniae* and *P. aeruginosa* isolates

Age range (years)	No. of	Percentage of isolates from:			
	patients	ICU (%)	Non-ICU (%)	Respiratory (%)	Blood (%)
18–30	210	37	63	63	37
31–40	166	39	61	59	41
41–50	244	44	56	50	50
51–60	470	42	58	50	50
61–70	597	65	35	51	49
>70	773	34	66	43	57
Total	2460	45	55	50	50

**Table 2:** MIC profile of AMK and comparators for isolates of *E. coli* and *K. pneumoniae*

Isolates	Antimicrobial agent	Range	Modal	MIC_50_	MIC_90_	%S
All isolates	AMK	≤0.5–>128	2	4	16	96
n = 1646	ATM	≤0.06–>64	0.06	0.125	32	85
	FEP	≤0.06–>64	0.06	0.06	32	87
	CAZ	≤0.06–>64	0.25	0.25	64	85
	C/T	≤0.06–>64	0.25	0.25	1	94
	CRO	≤0.06–>64	0.06	0.06	128	84
	CIP	≤0.015–>16	0.015	0.06	32	74
	IPM	≤0.06–>64	0.25	0.25	1	96
	MEM	≤0.06–>64	0.06	0.06	0.06	96
	TZP	≤0.25–>256	2	4	32	88
	TOB	≤0.06–>64	0.5	1	16	84
ESBL+isolates	AMK	0.5–>128	16	8	32	87
n = 173	ATM	≤0.06–>64	64	64	128	9
	FEP	≤0.06–>64	128	128	128	16
	CAZ	0.125–>64	128	64	128	14
	C/T	≤0.06–>64	0.5	1	8	79
	CRO	≤0.06–>64	128	128	128	5
	CIP	≤0.015–>16	32	32	32	16
	IPM	0.125–>64	0.25	0.25	1	94
	MEM	≤0.06–>64	0.06	0.06	0.125	95
	TZP	0.25–>256	4	8	256	62
	TOB	0.25–>64	32	16	64	39
FEP-R isolates	AMK	0.5–>64	8	8	64	79
n = 216	ATM	0.125–>64	128	64	128	6
	FEP	4–>64	128	128	128	0
	CAZ	0.5–>64	128	128	128	9
	C/T	0.125–>64	0.5	1	128	59
	CRO	≤0.06–>64	128	128	128	5
	CIP	≤0.015–>16	32	32	32	13
	IPM	0.06–>64	0.25	0.25	32	75
	MEM	≤0.06–>64	0.06	0.06	32	75
	TZP	0.25–>256	512	32	512	46
	TOB	0.25–>64	32	16	64	32
TZP-R isolates	AMK	0.5–>64	16	8	64	78
n = 191	ATM	≤0.06–>64	128	32	128	36
	FEP	≤0.06–>64	128	16	128	39
	CAZ	0.25–>64	128	64	128	33
	C/T	≤0.06–>64	128	1	128	56
	CRO	≤0.06–>64	128	128	128	35
	CIP	≤0.015–>16	32	32	32	34
	IPM	0.125–>64	0.25	0.5	32	70
	MEM	≤0.06–>64	0.06	0.06	32	71
	TZP	32–>256	512	256	512	0
	TOB	0.025–>64	32	16	64	39

*AMK* amikacin; *ATM* aztreonam; *FEP* cefepime; *CAZ* ceftazidime; *C/T* ceftolozane/tazobactam; *CRO* ceftriaxone, *CIP* ciprofloxacin; *IPM* imipenem; *MEM* meropenem; *TZP* piperacillin/tazobactam; *TOB* tobramycin

Of the 1646 *E. coli* and *K. pneumoniae* collected, 173 isolates were confirmed to be ESBL positive. Of these ESBL isolates, 87 % were found to have MICs ≤16 mg/L to AMK. When considering only the Enterobacteriaceae confirmed ESBL positive isolates the rank order susceptibility of the conventional agents was as follows: MEM 95 %, IPM 94 %, AMK 87 %, C/T 79 %, TZP 62 %, TOB 39 %, FEP 16 %, CIP 16 %, CAZ 14 %, ATM 9 % and CRO 5 %. For AMK 87 % of these organisms had MICs ≤16 mg/L, 9 % 32 mg/L and 2 % ≥64 mg/L. Of the total Enterobacteriaceae isolates collected three *E. coli* and 37 *K. pneumoniae* were carbapenemase-producing as defined by the CarbaNP test. The susceptibility of AMK, CIP and TOB for these isolates were 53, 15 and 10 %, respectively. All of the other agents had <9 % susceptibility. For AMK 53 % of these organisms had MICs ≤16 mg/L, 15 % 32 mg/L and 29 % 64 mg/L and 3 % 256 mg/L.

Hospitals provided 814 nosocomial blood and respiratory isolates of *P. aeruginosa*. Rank order % susceptibility was as follows: AMK 95 %, C/T 95 %, TOB 90 %, CAZ 74 %, FEP 73 %, MEM 71 %, CIP 68 %, ATM 67 %, TZP 67 % and IPM 62 % (Table 3). For AMK 95 % of these organisms had MICs ≤16 mg/L, 2 % 32 mg/L, 2 % 64 mg/L and 1 % ≥128 mg/L (Fig. 1). Comparing AMK against MEM resistant isolates (n = 236), 91 % of these organisms had MICs ≤16 mg/L, 6 % 32–64 mg/L and 3 % ≥128 mg/L. Comparing AMK against FEP resistant isolates (n = 222), 89 % of these organisms had MICs ≤16 mg/L, 9 % 32–64 mg/L and 2 % ≥128 mg/L. Comparing AMK against TZP resistant isolates (n = 267), 91 % of these organisms had MICs ≤16 mg/L, 7 % 32–64 mg/L and 1 % ≥128 mg/L. Like the Enterobacteriaceae, *P. aeruginosa* isolates from blood had a higher (3–14) % susceptibility for all agents tested than the respiratory isolates. The susceptibility profile of ATM, FEP, CAZ, IPM, MEM and TZP was 10 to 14 % higher from patients with blood cultures.

**Table 3:** MIC profile of AMK and comparators for isolates of *P. aeruginosa*

Isolates	Antimicrobial agent	Range	Modal	MIC_50_	MIC_90_	%S
All isolates	AMK	≤0.5–>64	4	8	16	95
n = 814	ATM	≤0.06–>64	4	8	32	67
	FEP	≤0.06–>64	2	4	32	73
	CAZ	0.125–>64	2	4	64	74
	C/T	≤0.06–>64	0.5	0.5	4	95
	CIP	≤0.015–>16	0.125	0.25	16	68
	IPM	≤0.06–>64	1	2	16	62
	MEM	≤0.06–>64	0.5	1	16	71
	TZP	≤0.25–>256	8	8	256	67
	TOB	≤0.06–>64	0.5	1	8	90
MDR+isolates	AMK	0.5–>64	8	8	32	87
n = 116	ATM	0.25–>64	128	32	128	19
	FEP	2–>64	32	32	128	13
	CAZ	2–>64	128	64	128	16
	C/T	0.5–>64	1	2	32	78
	CIP	2–>64	32	16	32	11
	IPM	0.5–>64	32	16	32	11
	MEM	0.25–>64	16	16	64	17
	TZP	4–>256	256	256	512	6
	TOB	0.125–>64	1	4	128	52
MEM-R isolates	AMK	0.5–>64	8	8	16	91
n = 236	ATM	0.25–>64	32	16	64	35
	FEP	1–>64	16	16	64	42
	CAZ	0.5–>64	128	16	128	47
	C/T	0.25–>64	1	1	8	89
	CIP	0.06–>16	32	4	32	35
	IPM	0.25–>64	16	16	32	11
	MEM	4–>64	16	16	32	0
	TZP	4–>256	32	32	512	36
	TOB	≤0.06–>64	1	1	64	74
FEP-R isolates	AMK	0.5–>64	8	8	32	89
n = 222	ATM	0.25–>64	32	32	128	23
	FEP	16–>64	16	32	64	0
	CAZ	2–>64	128	32	128	26
	C/T	0.25–>64	1	2	8	85
	CIP	0.06–>16	32	4	32	39
	IPM	0.5–64	16	8	32	37
	MEM	≤0.06–>64	16	8	32	38
	TZP	1–>256	256	128	512	20
	TOB	0.25–>64	1	1	64	76
TZP-R isolates	AMK	0.5–>64	8	8	16	91
n = 267	ATM	0.25–>64	32	32	128	27
	FEP	1–>64	16	16	64	34
	CAZ	0.5–>64	128	32	128	31
	C/T	0.25–>64	1	2	8	88
	CIP	≤0.015–>16	32	2	32	46
	IPM	0.125–>64	32	8	32	39
	MEM	0.125–>64	16	4	32	44
	TZP	32–>256	32	128	512	0
	TOB	0.25–>64	1	1	64	78

*AMK* amikacin; *ATM* aztreonam; *FEP* cefepime; *CAZ* ceftazidime; *C/T* ceftolozane/tazobactam; *CIP* ciprofloxacin; *IPM* imipenem; *MEM* meropenem; *TZP* piperacillin/tazobactam; *TOB* obramycin

Fourteen percent (n = 116) of the *P. aeruginosa* population was defined as MDR. In this subset of MDR isolates, rank order %S was as follows: AMK 87 %, C/T 78 %, TOB 52 %, ATM 19 %, MEM 17 %, CAZ 16 %, FEP 13 %, IPM 11 %, CIP 11 % and TZP 6 %. While the MIC_90_ was 32 for AMK, 23 % of these organisms had MICs ≤4 mg/L, 38 % 8 mg/L, 26 % 16 mg/L 5 % 32 mg/L, 4 % 64 mg/L, 3 % 256 mg/L and 1 % ≥512 mg/L. Of note 2 *P. aeruginosa* isolates were found to have an AMK MIC of 65,536 mg/L.

The AMK distribution for *S. maltophilia* (n = 45) was 29 % of these organisms had MICs ≤16 mg/L, 16 % 32 mg/L, 9 % 64, 24 % 128 mg/L, 15 % 256 mg/L and 7 % ≥512 mg/L.

To determine if the susceptibility of these organisms are influenced by the source of infection and location of the patient, we evaluated the % susceptibility of ICU versus Non-ICU patients. The Enterobacteriaceae had no more than a 2 % difference in % susceptibility between the ICU (n = 539) and Non-ICU (n = 1106) patients. For the *P. aeruginosa*, we noted differing susceptibility profiles derived from the ICU and non-ICU setting. The susceptibility profile of ATM, FEP, CAZ, IPM, MEM and TZP was 5–11 % lower from patients in the ICU. In contrast, ≤3 % difference in was observed between the potency of ICU and non-ICU *P. aeruginosa* for AMK, C/T, CIP and TOB.

## Discussion

Hospital-acquired pneumonia (HAP) and ventilator-associated pneumonia (VAP) are among the most frequent nosocomial infections encountered in the ICU and are responsible for increases in length of stay, mortality and morbidity in critically ill patients. *P. aeruginosa,* along with the Enterobacteriacae, *E. coli* and *K. pneumonia* represent a substantial proportion of HAP and VAP infections. The use of empirical broad spectrum antibiotics for the treatment of pneumonia has led to an increase in antimicrobial resistance along with the development of multidrug resistant Gram-negative organisms.

Appropriate antibiotic exposure at the site of infection is an important component for therapy to be clinically effective. Epithelial lining fluid (ELF) is considered to be the site of infection for pneumonia and achieving sufficiently high drug concentrations in ELF are critical for treating pneumonia. The rationale for inhaling antibiotics is to maximize drug delivery to the target site of infection (ELF in the case of pneumonia) and limit the potential for systemic side effects. The uses of inhaled antimicrobials have a long history in the treatment of lower respiratory tract infections specifically in patients with cystic fibrosis [11]. Due to the increasing prevalence of resistant Gram-negative organisms in VAP causing difficult to treat pneumonia along with the limited lung concentrations that can be achieved with standard parentally given antibiotics there has been an increased interest in the use of inhaled antimicrobials such as inhaled amikacin for respiratory tract infections such as in HAP and VAP [12]. In a recently published study, So et al. [6] investigated the in vitro pharmacodynamics of human simulated ELF concentrations of inhaled amikacin against variety of phenotypically diverse susceptible and non-susceptible *K. pneumoniae* and *P. aeruginosa* isolates and showed rapid bactericidal activity against isolates with an amikacin MICs of ≤256 mg/L.

The high overall susceptibility profile of amikacin against these Gram-negative organisms of interest in our current study appears to represent the sustained in vitro potency of the compound when compared to two previously reported surveillance programs [13, 14]. The INFORM study collected 7062 clinical *P. aeruginosa* isolates from Europe, Asia/South Pacific, Latin America and Middle East/Africa [13]. Similar to our data the INFORM global surveillance program found amikacin to have a high susceptibility (89.4 %) against the *P. aeruginosa* isolates tested. Moreover, the authors also reported similar (±6 %) susceptibility profiles for ceftazidime, cefepime, piperacillin-tazobactam and meropenem to that in our current study.

The SMART surveillance program collected intra-abdominal isolates of *E. coli, K. pneumoniae*, *K. oxytoca* and *P. mirabilis* [14]. *E. coli* (n = 434) maintained high susceptibilities to amikacin of >99 %, while *K. pneumoniae* (n = 231) had 93.5 %. Both organisms had ≥92 % susceptibility to imipenem and ertapenem. In the SMART program amikacin retained the highest susceptibility to MDR Enterobacteriaceae of all the agents tested.

## Conclusions

In this study we defined the phenotypic profile of amikacin against 2460 blood and respiratory nosocomial isolates implicated in ICU based pulmonary infections collected from 50 US hospitals. AMK demonstrated a high level of activity to combat infections caused by the Enterobacteriacae, *E. coli* and *K. pneumoniae* including those strains producing ESBLs. When considering *E. coli* and *K. pneumoniae* our study found amikacin to have an MIC_50_ and MIC_90_ of 4 and 16 mg/L, respectively. Moreover, 96 % of organisms had a MIC of ≤16 mg/L and nearly all (99 %) organisms had MICs ≤64 mg/L despite concomitant β-lactam or fluoroquinolone resistance. In a study conducted by Sader et al. [15] analyzing the in vitro activity of amikacin against isolates gathered from patients hospitalized with pneumonia including VAP, across 62 US hospitals, the MIC_50_ and MIC_90_ was reported to be 1 and 32 mg/L, respectively, against *K. pneumoniae.* In a study investigating *E. coli* isolates across 66 Canadian medical centers the reported amikacin MIC_50_ and MIC_90_ were ≤2 and 4 mg/L, respectively [16].

For *P. aeruginosa*, our study found amikacin to have an MIC_50_ and MIC_90_ of 8 and 16 mg/L, respectively. Against these isolates 95 % of organisms had amikacin MICs of ≤16 mg/L, moreover nearly all (97 %) organisms have MICs ≤32 mg/L. Similarly when compared to the Sader et al. [15] study, *P. aeruginosa* isolates from 62 US hospitals, amikacin was reported to have an MIC_50_ and MIC_90_ of 4 and 8 mg/L, respectively. Another study analyzing similar data from 127 isolates from Canadian hospitals, reported MIC_50_ and MIC_90_ of 4 and 16 mg/L, respectively [17].

With respect to resistant Gram-negative bacteria, our study demonstrated that amikacin maintained potent activity against *P. aeruginosa* MDR organisms. In regards to ESBLs this study showed that AMK was a potent antimicrobial with a high level of activity against these MDR isolates as 87 % had an MIC of ≤16 mg/L. Against MDR *P. aeruginosa* isolates, amikacin was the most potent antibiotic tested as 87 % of resistant isolates were considered susceptible (i.e., MIC of ≤16 mg/L).

The current surveillance study which incorporated a large number of US hospitals demonstrated the high potency of amikacin against contemporary isolates of *E. coli*, *K. pneumoniae, P. aeruginosa* originating from a blood or respiratory source. Moreover, despite resistance to representative agents from the β-lactam and fluoroquinolone classes, AMK maintained high susceptibility for all organisms. As a result of low MIC’s and high achievable lung concentrations of approximately 5000 mg/L, Amikacin Inhale has a potential adjunctive role in the management of bronchopulmonary infections caused by *P. aeruginosa, E. coli* and *K. pneumoniae* in the intubated patient. While Amikacin Inhale appears to be a viable adjunctive therapy, patients should also receive appropriate systemic antimicrobial therapy based on local susceptibilities or when available the susceptibility profile of the patient specific isolate(s).

## Abbreviations

HAP: hospital-acquired pneumonia
VAP: ventilator-associated pneumonia
ICU: intensive care unit
*P. aeruginosa*: *Pseudomonas aeruginosa,*
*E. coli*: *Escherichia coil*
*K. pneumoniae*: *Klebsiella pneumoniae*
CT: Connecticut
US: United States
AMK: amikacin
ELF: epithelial lining fluid
*S. maltophilia*: *Stenotrophomonas maltophilia*
CLSI: Clinical Laboratory Standards Institute
MICs: minimum inhibitory concentration
ATM: aztreonam
FEP: cefepime
CAZ: ceftazidime
C/T: ceftolozane/tazobactam
CRO: ceftriaxone
CIP: ciprofloxacin
IPM: imipenem
MEM: meropenem
TZP: piperacillin/tazobactam
TOB: tobramycin
MO: Missouri
FDA: food and drug administration
MDR: multidrug resistant
ESBL: extended spectrum β-lactamases
*K. oxytoca*: *Klebsiella oxytoca*
*P. mirabilis*: *Proteus mirabilis*
